# Results from the Medical School Physical Activity Report Card (MSPARC) for a Thai Medical School: a mixed methods study

**DOI:** 10.1186/s12909-018-1408-7

**Published:** 2018-12-04

**Authors:** Apichai Wattanapisit, Surasak Vijitpongjinda, Udomsak Saengow, Waluka Amaek, Sanhapan Thanamee, Prachyapan Petchuay

**Affiliations:** 1 School of Medicine, Walailak University, Tha Sala, Nakhon Si Thammarat, 80161 Thailand; 2 Center of Excellence in Health System and Medical Research, Walailak University, Tha Sala, Nakhon Si Thammarat, 80161 Thailand; 3 School of Architecture and Design, Walailak University, Tha Sala, Nakhon Si Thammarat, 80161 Thailand; 4 Tha Sala Hospital, Tha Sala, Nakhon Si Thammarat, 80160 Thailand

**Keywords:** Medical education, Medical school, Medical student, Physical activity, Report card, Surveillance

## Abstract

**Background:**

Data systems for surveillance and monitoring are essential to develop understanding of the levels of physical activity (PA) occurring at the population levels. To comprehensively understand PA in medical schools, a suitable surveillance tool might be useful to present and monitor key PA-related metrics. This study investigated PA-related metrics in a Thai medical school and summarised the findings using a newly developed tool-the Medical School Physical Activity Report Card (MSPARC).

**Methods:**

A mixed methods study was carried out at three campuses of a medical school in Southern Thailand. Data analysis included descriptive statistics and qualitative reviews. All 285 medical students from a medical school were the target population. The prevalence of PA (≥ 150 min/week of moderate- to vigorous-intensity PA) and sedentary behaviours (SB) (≥ 8 h/day of sedentary time) among medical students were analysed using data from a self-administered questionnaire. Usage patterns, quality, and accessibility of walkable neighbourhoods; bicycle facilities; and recreational areas were assessed. PA promotion programmes, education, and investment related to PA promotion were reviewed from the school documents.

**Results:**

Of 279 participants, 138 (49.5%) met PA recommendation, but 71.7% met criteria for SB. Male students were more active (61.8 vs. 42.4%) and less sedentary (65.7 vs. 75.1%) than female students. Bicycle facilities were rated as having the lowest quality and accessibility among PA-related facilities. Most PA promotion programmes were sports clubs and sport competitions. A total of 25 h of PA education was taught throughout the entire curriculum, which provided minimal PA counselling training. The school invested 2136.14 Baht/student/year (US $64.34) in PA promotion, or 2.4% of the annual tuition. The MSPARC presented the summary of the findings by using simple symbols, infographics, and short texts.

**Conclusions:**

To increase PA and decrease SB among medical students, there is a need to improve the quality and accessibility of the built environment as well as the natural environment, so as to establish health promoting policies. PA counselling training is required to develop the medical students’ essential skills and awareness for future practices. Monitoring and subsequent surveillance of PA in medical school are needed.

## Background

Physical activity (PA) has well-documented evidence in promoting health and preventing and treating diseases [1–4]. One of the global health and sustainable development goals is promoting PA in population levels [5]. Data systems for surveillance and monitoring are essential to develop understanding of the levels of PA occurring at the population levels [6, 7]. Suitable surveillance methods might be useful to present and monitor PA-related metrics. There are several surveillance methods used in different populations and settings [6–11]. Given the setting of this study, a medical school, the benefits and weaknesses of specific approaches should be considered.

In Thailand, there are twenty-three medical schools and more than two thousand medical students graduate from those medical schools each year [12]. It is important to increase and monitor PA in medical students. Since good PA habits are beneficial for their long-term health and wellness, and are a factor associated with providing appropriate lifestyle counselling to their patients [13, 14]. However, a previous study shows PA prevalence among medical students in Southern Thailand is lower than in the Thai adult population (49.5 vs. 70%) [15, 16]. This reflects a challenge for Thai medical schools to promote healthy lifestyles for their students.

There is a need to understand PA habits and determinants of PA among medical students. The evidence supports that environments and policies are the determinants of PA in various settings [17, 18]. For example, larger campus per student is associated with increased PA [19]. Therefore, understanding the environmental limitations of the facilities available for PA and key institutional policies related to encouragement PA are necessary in a particular setting [20, 21]. PA teaching in the medical school curricula is a specific indicator of the academic emphasis placed by the institution [22–24]. As a consequence, appropriate surveillance of ongoing PA habits by medical students requires administrative assessments to follow the relevant metrics for PA and to identify good strategies for promoting regular PA. To address that goal, a suitable surveillance tool appropriate for medical students might be useful to present and monitor key PA-related metrics.

To comprehensively survey the levels of PA of students in medical schools, including gaining an understanding of the medical school profile, the prevalence of PA overall and in sub-populations of medical students, the availability of PA-related facilities, and the applicable institutional policies is a significant research question. Therefore, the researched team developed the Medical School Physical Activity Report Card (MSPARC) in 2017. The MSPARC is a specific PA report card designed for medical schools to document (1) the fundamental profile of the medical school, (2) the student PA habits and sedentary behaviours (SB), (3) PA-related environments, (4) policies related to PA, and (5) the PA surveillance plan [25]. The aims of this paper are to describe the methods used to collect data for the first in-depth PA surveillance at the School of Medicine, Walailak University, Thailand, and to summarise the results using the MSPARC.

## Methods

This section presents a brief statement of the research methods. The protocol paper published elsewhere provides full details of the methodology [25].

### Study design

The mixed methods study was carried out from January to September 2017. The study consisted of two parts-a quantitative design (survey study) and a qualitative design (case study).

### Setting and participants

The study was conducted at three campuses of the School of Medicine, Walailak University, Thailand. A total of 285 Thai medical students were enrolled in academic year 2017. All preclinical students (years, 1–3) were at Nakhon Si Thammarat main campus and clinical students (years, 4–6) did their clinical rotations at Trang Hospital or Vachira Phuket Hospital (clinical campus).

### Data collection and analysis

The quantitative data were collected by the self-administered questionnaires and were transferred to a data entry software, EpiData version 3.1 (EpiData Association, Denmark). Quantitative data analysis was operated by using descriptive statistics. Statistical analysis was operated by using RStudio version 0.99.491 with epical package (RStudio, USA). The policies related to PA were collected by reviewing from the school document and all qualitative data were independently analysed by two researchers.

#### General information

The recent university and/or faculty documents were reviewed to identify the land area of the main campus, the number of students, and annual tuition.

#### People (student data)

Information on PA and sedentary time was reanalysed from the previous survey in 2016, that used the Global Physical Activity Questionnaire (GPAQ) to collect data [16]. The GPAQ was considered as a valid and reliable instrument in assessing PA compared to accelerometer and the International Physical Activity Questionnaire (IPAQ) [26, 27]. The GPAQ was available in Thai version and widely used for population based surveys in Thailand [28, 29]. The prevalence of PA was calculated by dividing the number of participants who met the recommended aerobic PA levels (at least 150 min/week of moderate-intensity aerobic PA or 75 min/week of vigorous-intensity aerobic PA or an equivalent combination of moderate- and vigorous-intensity aerobic PA) [30] by the total number of participants. The prevalence by sex and educational level (preclinical and clinical levels) were also calculated. The prevalence of SB (≥8 h per day of sedentary time) [31, 32] were computed for the entire group of medical students, for students of each sex, and for the groups in the preclinical and clinical years.

#### Places (campus facilities)

The data on PA-related places at the main campus reported by the preclinical students by a self-administered questionnaire, and focused on activity in three environments: walkable neighbourhoods, bicycle facilities, and recreational areas. The data regarding usage of places (frequency of facilities use for users and barriers to facilities for non-users) was described as frequencies and percentages. The mean score of overall quality and accessibility of each category of place was calculated from the self-rating scales (0 to 10).

#### Policies

The PA-promotion programmes for medical students, PA education, and investments related to PA promotion were independently reviewed and analysed from the available annual plans, reports and curriculum by two researchers (SV and US). Any differences in the interpretations of the two analysts were discussed and the appropriate conclusion confirmed by the research team.

### Medical school physical activity report card

The MSPARC was created by using a computer programme, Adobe Illustrator version CC 2017 (Adobe Systems Incorporated, USA), to present the summary of the PA surveillance using simple symbols, infographics, and short texts.

## Results

### General information

The School of Medicine is located at the main campus of Walailak University in Nakhon Si Thammarat, Thailand. The campus covers 15.35 km^2^ of land. In academic year 2017, there were 285 medical students. The tuition was 90,000 Baht/year (US $2710.84).

### People

A total of 279 medical students (97.9%) with mean age 20.93 ± 1.82 years participated in the survey on PA and SB in 2016. The majority of participants were female (63.4%, *n* = 177) and preclinical students (50.5%, *n* = 141). About half of the students (49.5%, *n* = 138/279) reported that they were physically active. Males were more active than females (61.8%, *n* = 63/102 vs. 42.4%, *n* = 75/177, *p* < 0.05). Preclinical students reported they were more active than the students doing clinical rotations (58.2%, *n* = 82/141 vs. 40.6%, *n* = 56/138, *p* < 0.05). Male preclinical students were the most active (72.7%, *n* = 40/55). In contrast, the lowest prevalence of PA was in female clinical students (36.3%, *n* = 33/91).

The prevalence of SB was 71.7% (*n* = 200/279). Male clinical students had the lowest prevalence of SB (44.7%, *n* = 21/47). The most sedentary group was female preclinical students (90.7%, *n* = 78/86) (Table 1).

**Table 1:** Physical activity and sedentary behaviours among medical students

	PA (≥ 150 min/week of MVPA) n (%)	SB (≥ 8 h/day of sedentary time) n (%)
Total (*n* = 279)	138 (49.5)	200 (71.7)
Male (*n* = 102)	63 (61.8)	67 (65.7)
Female (*n* = 177)	75 (42.4)	133 (75.1)
Preclinical students (n = 141)	82 (58.2)	124 (87.9)
Male (*n* = 55)	40 (72.7)	46 (83.6)
Female (*n* = 86)	42 (48.8)	78 (90.7)
Clinical students (n = 138)	56 (40.6)	76 (55.1)
Male (*n* = 47)	23 (48.9)	21 (44.7)
Female (*n* = 91)	33 (36.3)	55 (60.4)

*MVPA* moderate- to vigorous-intensity physical activity, *PA* physical activity, *SB* sedentary behaviours

### Places

Of the preclinical students in 2017, 143 (99.3%) completed the questionnaires regarding the locations where they participated in PA-related activities. Most students (91.6%, *n* = 131/143), used recreational areas on campus and more than half (55.7%, *n* = 73/131) used these facilities 3 to 4 days/week. The survey indicated that the quality of recreational areas was rated at 6.1 out of 10 and the accessibility of the facilities was rated 6.7 of 10.

Walkable neighbourhoods were accessed by 61.5% (*n* = 88/143) of the students. About half, 47.7% (*n* = 42/88) reported that they utilised walkable neighbourhoods 3 to 4 days/week. The most common barrier to walking cited by the non-users was that access to these areas was unavailable or inconvenient. The quality and accessibility of the walkable neighbourhoods to the students were rated at 5.3/10 and 6.3/10, respectively.

Only 16 of 143 medical students (11.2%) reported using bicycles. More than half of the non-cyclists (58.3%, *n* = 74/127) stated that the bicycle facilities provided on campus were either unavailable or inconvenient, and about a quarter (23.6%, *n* = 30/127) of the students raised safety concerns. Both the quality (4.8/10) and accessibility (4.9/10) of the bicycle facilities were scored at lowest levels reported for any of the PA-related activities (Table 2).

**Table 2:** Physical activity-related places survey (*n* = 143)

	Walkable neighbourhoods	Bicycle facilities	Recreational areas
Usage			
Number of users, n (%)	88/143 (61.5)	16/143 (11.2)	131/143 (91.6)
Frequency (users), n (%)			
Sometimes (1–2 days/week)	32/88 (36.4)	8/16 (50.0)	45/131 (34.4)
Often (3–4 days/week)	42/88 (47.7)	4/16 (25.0)	73/131 (55.7)
Always (5–7 days/week)	14/88 (15.9)	4/16 (25.0)	13/131 (9.9)
Barriers (non-users), n (%)			
Not interested/dissatisfied	6/55 (10.9)	24/127 (18.9)	6/12 (50.0)
Unavailable/inconvenient	45/55 (81.8)	74/127 (58.3)	4/12 (33.3)
Unsafe	12/55 (21.8)	30/127 (23.6)	6/12 (50.0)
Other	5/55 (9.1)	34/127 (26.8)	1/12 (8.3)
Quality (0–10)			
Mean (SD)	5.3 (1.8)	4.8 (2.1)	6.1 (1.8)
Accessibility (0–10)			
Mean (SD)	6.3 (1.9)	4.9 (2.3)	6.7 (1.9)

*SD* standard deviation

### Policies

The PA promotion programmes for medical students were classified into three categories as following: sports clubs (3 programmes) - yoga club, football (soccer) club, and badminton club; sport competitions (3 programmes) - Thai Medical School Sport Competition (Syringe Games), Health Sciences Games, and Sport Days (among campuses); and other (1 programme) - Dance Therapy (1-day activity).

PA education was provided in both the preclinical and clinical years. Basic knowledge of PA, including an overview of exercise physiology, cardiovascular adjustment to exercise, energy for PA, exercise for chronic diseases, therapeutic exercise, and sports injuries, were the main components of PA education in the curriculum (18 h). Education on PA and public health consisted of principles of PA for community, and health promotion and behavioural changes, took 7 h in the curriculum. PA counselling was not included in the medical curriculum (Table 3).

**Table 3:** Physical activity education (hours)

	Total	Preclinical years	Clinical years
Basic knowledge of PA	18	14	4
PA and public health	7	4	3
PA counselling	0	0	0
Total	25	18	7

*PA* physical activity

The institution spent 608,800 Baht/year (US $18,337.35) on PA promotion for medical students. The per capita investment on PA promotion was 2136.14 Baht/student/year (US $64.34) and the ratio of per capita investment to annual tuition fee was 0.024 (2.4%).

### Medical school physical activity report card

**Fig. 1:**
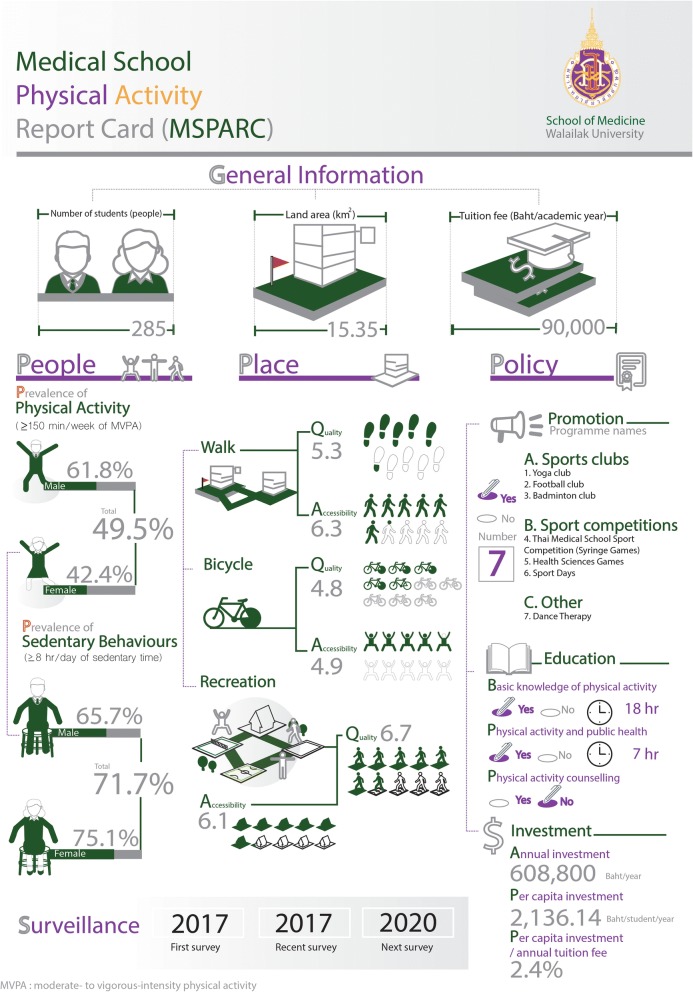
Summary of the 2017 physical activity surveillance in the Medical School Physical Activity Report Card (MSPARC)

Figure 1 shows the summary of the 2017 PA surveillance. The MSPARC illustrates general information such as land area, number of students and tuition fee; people—prevalence of PA and SB; places—quality and accessibility of walkable neighbourhoods, bicycle facilities, and recreational areas; policies—PA promotion programmes for medical students, education metrics and investment related to PA; and surveillance scheme—first, recent, and next surveys.

## Discussion

This is the first study of PA surveillance to explore comprehensive behavioural metrics in the students of a Thai medical school. The results indicated that 50.5% of the medical students were physically inactive, and 71.7% of them spent 8 h/day or more in sedentary activities. Commuting to the medical school by bicycle was not popular among the students, reportedly because the campus bicycle facilities were considered unavailable or inconvenient and unsafe. The medical school utilised 2.4% of the student tuition payments for promotion of PA, which included sports clubs and sport competitions. According to the available records, the 6-year curriculum devoted 25 h to education to provide some basic knowledge of the medical aspects of PA, as well as the role of PA in public health programmes.

The findings showed that the medical students at an institution in Southern Thailand engage in less PA than members of the Thai adult population [15]. In contrast, previous studies carried out in both the US and Thailand reported that medical students were more active than the general population [33, 34]. These different outcomes may reflect a lack of a supportive environment and policies present at the institution. As noted, transport involving PA, walking, and cycling, was not a popular activity for the students in terms of quality and accessibility. Although most medical students reported that they accessed the available recreational facilities, the quality and accessibility was suboptimal. According to the current policies, PA promotion for medical students had a low priority and it was considered to be an insignificant issue. The medical school invested very limited resources in this aspect of medical education, and the PA promotion programmes mainly focused on students who were interested in participating in sports through sports clubs and competitions.

Preclinical students were more physically active than the students in their clinical years. Surprisingly, the preclinical students were more likely to report SB than the students doing their clinical training. This seemingly paradoxical finding may be explained by two factors. First, the preclinical students spent much of their day in lectures, and thus would be sedentary during their hours in school. However, they had more flexible schedules and free time compared to students doing clinical rotations [35]. Therefore, they had more leisure time for activities that could involve recreational PA. Second, the differences of academic activities between the preclinical and clinical years contributed to the different sedentary times. Although the preclinical students spent most of their academic periods in classrooms, the students doing clinical years would typically be physically engaged while making ward rounds on their patients, or in the hospital clinics or surgical rotations. Although students doing clinical rotations might do more walking with their hospital activities, the extent of ambulation during ward rounds was still considered light-intensity PA [36–38]. which was not accounted for by the questionnaire [39]. In terms of gender differences, male medical students were more likely to be physically active than female students. This trend was also found from other medical schools in different countries [40, 41]. This phenomenon might occur because of differences of psychosocial factors and influences on PA between males and females. Edwards et al. summarise that males have higher levels of self-efficacy towards PA, greater levels of social support, and stronger motivation (e.g., challenge, strength, competition, and social recognition) [42].

The present study found that Walailak University provided more PA education (25 h) across the medical school programme than medical school curricula in the UK (4.2 h), [24] US (8.1 h) [22], or Australia (12.3 h) [23]. The Medical Council of Thailand requires that all Thai medical schools teach about health promotion, including exercise [43]. However, it does not identify any specific topics about PA education. In contrast, from a qualitative analysis, this study classified the PA education in the curriculum into three different topics. The findings could be summarised that most of the PA education provided did not address patient counselling about the benefits and practices of PA. Worldwide, PA counselling has been reported as being inadequately addressed in medical education [44, 45].

The MSPARC provides concise information and can be read at a glance. The simplicity of the tool might lead to more effective communication to public and the relevant parties. Similarly, a single-slide infographic provided summary of financial aspects, PA and sport participation among third level students in Ireland is also used to represent the concise data [46]. The MSPARC, specifically, was developed as a tool to present and monitor PA-related metrics in medical schools. The MSPARC can be adopted for surveillance and is adjustable for use at other medical schools or institutions. For example, finding the prevalence of PA and SB can be based on the whole population, or it can be done by a randomised sampling protocol from the entire student body. Using self-rating scales to grade the quality and accessibility of both the built and natural environments that are available to students, MSPARC is not limited by differences of the facilities and features of the different neighbourhoods around the medical schools. The results in the MSPARC are informative and can be a benchmark among medical schools.

### Strengths and limitations

The strengths of the study included the comprehensiveness of the PA-related metrics. The various indicators could represent specifically holistic data to the medical school. To the best of our knowledge, this is the first study performed at a medical school to gather specific information on PA-related policies, including PA promotion, education, and institutional investment. In addition, the study design surveyed PA-related metrics and developed the MSPARC simultaneously. We found that the MSPARC was a suitable tool to present the identified metrics. There were some limitations in this study. First, the study was conducted in a Thai medical school to understand a particular setting, which could not be generalisable to other medical schools. Second, the prevalence of PA and SB was recapitulated from the previous study in 2016 because of a lack of resources that otherwise were primarily studied in 2017. Third, this study presented PA and SB at a particular moment which could vary in different seasons. Fourth, the use of self-reported data provided the subjective data. Lastly, the new tool, MSPARC, had not been appraised previously for validity, effectiveness, and feasibility.

### Recommendations

There is a need to present information regarding low PA and high SB of medical students at the institution to all the relevant parties to increase public awareness of these health behaviours, and the possible impact they may have on the long-term health of the trainees. Strategies to promote PA and reduce SB among medical students should be prioritised and viewed as essential elements of training, to foster better health of the students and thus also patient care [13, 47].

As they were the most physically inactive and highly sedentary, major new efforts should be focused on health promotion for the female medical students. Among the preclinical students, reduction of time spent sitting is one strategy that might be emphasised to lower the risks of disease. For students during the clinical years, PA promotion programmes to increase amount of moderate- to vigorous-intensity PA are needed, to begin to inculcate good lifestyle habits.

Since medical students during the clinical years were busy with hospital activities, one strategy to decrease SB is to use adjustable height desks to allow the medical students to write their clinical notes or type them into the hospital computer system/electronic medical record while standing [48, 49]. Another alternative strategy is to decrease time spent sitting and increase energy expenditure during their activities. The senior staff can conduct clinical rounds and other group teaching activities while moving to multiple locations on different floors, so as to be able to incorporate climbing up and down the stairs. This is particularly useful as mandated PA [50].

PA promotion programmes should be expanded beyond participation in organised sports. The promotion programmes should contribute to increasing participation by the more sedentary and physically inactive students in daily life activities. Supportive actions, for instance, could include instituting a walking campaign and group exercises. The quality and accessibility of the built and natural environments on campus should be improved to be safe, accessible, and user friendly.

A cost-effective investment in PA promotion is a considerable issue and either increasing the budget for PA promotion or enhancing the coverage of PA promotion programmes would be required. In terms of medical education, formal training for PA counselling can be beneficial. By adding teaching sessions to the curriculum, the institution could enhance awareness and perhaps good attitude toward health promotion of these future doctors.

According to the ecological model, PA behaviours are influenced by multiple levels of factors, including, individual, interpersonal, organisational, societal, and community factors [51]. Moreover, the interactions among each level of factors influence individuals and subsequent behaviours [51]. To increase PA and reduce SB needs multilevel interventions. Any interventions involve multiple levels of factors are more effective than single-level interventions [52]. Medical schools should focus on multilevel interventions to fight the low levels of PA and high levels of sedentariness of medical students as a whole system. A system-based approach with the actions of policymakers and individuals is needed. For example, if policymakers announce an active lifestyle as an organisational culture, medical students are more likely to adjust their lifestyles being more active. Additionally, the presentation of the MSPARC should be used as a scoreboard to notice the problems and inform all the relevant parties to achieve the goals to increase PA and reduce SB among medical students. Finally, the MSPARC should be assessed after the implementation.

## Conclusions

More than half of medical students are physically inactive and sedentary during the years they are in academic and/or clinical training. To promote PA in medical schools, there is a crucial need to improve the quality and accessibility of the built environment as well as the natural environment, so as to establish health promoting policies and procedures. Better promotion of PA for sedentary and physically inactive medical students is needed, encouraging both increased sports activities and non-sports behaviours. Moreover, development of better clinical training for patient counselling about the benefits PA for long-term health and fitness is required to develop the medical students’ essential skills and awareness for their future practices. Continuous monitoring and subsequent surveillance of PA in medical school are recommended. Furthermore, medical professionals should be role models for healthy living in their communities, and medical schools can be the prime movers to advocate for health-related public policies.

## Abbreviations

MSPARC: Medical School Physical Activity Report Card
PA: Physical activity
